# Using the Novel Musculoskeletal Emergency Medicine Assessment Tool: A Feasibility Study

**DOI:** 10.7759/cureus.61740

**Published:** 2024-06-05

**Authors:** Allison D Lane, Alexander J Tomesch, Anna Waterbrook, William Denq

**Affiliations:** 1 Emergency Medicine/Sports Medicine, University of Arizona College of Medicine - Tucson, Tucson, USA; 2 Emergency Medicine/Sports Medicine, University of Nebraska Medical Center, Omaha, USA

**Keywords:** resident assessment, emergency medicine assessment tool, musculoskeletal assessment tool, musculoskeletal assessment, musculoskeletal education, emergency medicine musculoskeletal assessment, resident education

## Abstract

Background and objective

While musculoskeletal (MSK) disorders account for a significant number of primary care and emergency department (ED) visits, there are widely recognized shortcomings and gaps in MSK education throughout medical training. Undergraduate medical education (UME) frequently fails to impart clinically relevant MSK knowledge, while many emergency medicine (EM) residency graduates report feeling unprepared to manage MSK complaints. Existing MSK assessments are not tailored to EM and may inaccurately assess specialty-specific MSK knowledge. The novel validated Musculoskeletal Emergency Medicine Assessment Tool (MEAT) holds great promise in standardizing EM MSK knowledge assessment. This trial of feasibility was conducted to assess the viability and practicality of using MEAT to evaluate MSK knowledge among incoming resident physicians in EM programs.

Methods

This feasibility study involved 21 incoming EM resident physicians from two programs at a single institution. MEAT was administered online during orientation, and demographic data and survey metadata were collected. UME MSK education details were obtained, and MEAT scores were analyzed.

Results

Participants reported no difficulties in accessing or understanding the 50-question online MEAT, resulting in a 100% response rate. The average pretest score for all interns was 29.9, with a median of 30. Most participants had documented UME MSK education, but curricular content varied widely. The participants took an average of 32 minutes to complete the assessment.

Conclusions

MEAT demonstrated successful implementation and high response rates, suggesting a high level of feasibility. The tool can be used to assess baseline MSK knowledge and ultimately track progression during residency with the potential for evaluating educational interventions once further validation studies have been performed. Further adoption of MEAT across multiple EM residency programs will help to enhance the tool's generalizability.

## Introduction

Musculoskeletal (MSK) disorders account for nearly 20% of primary care and emergency department (ED) visits annually ​[1]. Despite this prevalence, there are several documented lacunae in MSK education across all levels of medical training and practice ​[2-5]. Several studies have shown that undergraduate medical education (UME) often falls short in providing clinically relevant MSK knowledge, including diagnosis and management of common MSK conditions ​[2,3,6]. A survey revealed that only 54% of medical students felt their MSK education was adequate ​[7]. Furthermore, only 83% of medical schools require pre-clinical MSK education, and a mere 15% mandate an MSK clerkship ​[8]. This variability in UME MSK curricula underscores the challenge of defining essential foundational MSK education.

Moreover, emergency medicine (EM) residency graduates have reported feeling inadequately prepared to manage MSK complaints ​[9]. A national needs assessment of EM residencies in 2021 highlighted that most respondents felt their curriculum could be improved and expressed a desire to have a standardized MSK assessment specific to EM training ​[10]. Existing validated MSK examinations, such as the Freedman and Bernstein assessment (FB-MSK), were developed for medical students ​[2]​ and are potentially inadequate for EM residents due to their non-specialized nature and the subjective grading involved. Scoring on these assessments varies significantly based on the specialty of the test taker and who is grading the test ​[11-13]. Significant variability is noted in scores depending on the rotations a student participated in as well; e.g., those with an orthopedic rotation scored 60.3% compared with 45.1% for those without ​[11]. When testing residents, primary care trainees scored an average of 56.3%, EM residents 77.5%, and orthopedic residents 90%. This raises the concern that this tool may not be adequately relevant and effective for assessing MSK knowledge in non-orthopedic residents and other graduating medical students ​[3]. The MSK30 is another validated MSK assessment tool that uses multiple choice questions (MCQs) to mitigate the element of subjective grading, but it is recommended primarily for medical schools and primary care residencies ​[3]. Additionally, neither the FB-MSK nor the MSK30 have been described as being administered in an online format.

We recently developed and validated the Musculoskeletal Emergency Medicine Assessment Tool (MEAT), an MCQ-based tool focusing on MSK knowledge relevant to EM and designed for online administration and distribution ​[14]. To our knowledge, MEAT is the first assessment tool specifically validated for EM resident physicians. MCQ assessments like MEAT and MSK30 offer advantages such as immediate scoring and reduced grading subjectivity ​[2,3,14]. MEAT can be distributed through a variety of methods including e-mail, text messaging, or via a QR code. It can be formatted to be taken on a personal computer or handheld device. Although MCQ assessments such as MEAT have certain limitations, they are widely used in medical knowledge evaluations ​[3,15-17]. In addition, reports show that MCQ assessments are frequently administered online without any compliance or administration issues ​[16,17].

Using MEAT at various training stages enables EM residency programs to track the progression of EM-relevant MSK knowledge and evaluate the effectiveness of educational interventions, such as MSK rotations or workshops. However, there are no published trials of this assessment in EM residencies. In light of this, our feasibility study aims to assess incoming resident physicians at the onset of their graduate medical education (GME) using in-person electronic administration of MEAT.

## Materials and methods

This feasibility study was conducted in 2022 and involved all 21 incoming resident physicians from two categorical EM programs at a single institution. One program is affiliated with a level-one academic teaching hospital and the other program is associated with a level-four community hospital.

Using the Qualtrics platform, the MEAT (see Appendices) was incorporated into a survey format and distributed to participants via an anonymous link during intern orientation. This assessment was completed before any formal GME MSK training, ensuring scores reflected baseline knowledge. To ensure the integrity and confidentiality of the data, each participant was assigned a randomized identification number (rID). This identifier was used for tracking scores and making future comparisons without compromising anonymity. Upon completion of the assessment, individual scores were automatically transmitted to the administrator. One point was assigned to each question, for a total possible score of 50. 

Demographic data, details of the current GME program, UME location, degree type, and UME MSK curriculum information were collected during assessment administration (Table 1). Additionally, survey metadata, including the duration of each assessment, was captured for analysis. Summary statistics were computed using Microsoft Excel. 

**Table 1:** Demographic Data MSK: musculoskeletal; PGY: postgraduate year

Question	Answer
What institution are you from?	Academic campus, Community Campus
What year are you?	PGY-1, PGY-2, PGY-3
Where was your medical school located?	Midwest, Northeast, West, South
What type of medical degree do you have?	Allopathic, Osteopathic
What country did you attend medical school in?	(Specify)
Did you participate in any of the following rotations during medical school? Check all that apply	Physical Medicine and Rehabilitation, Orthopedics, Rheumatology, Sports Medicine, Other (specify)
Were musculoskeletal topics taught in your medical school's curriculum?	Yes or No
Were musculoskeletal topics taught in your medical school's curriculum during pre-clerkship?	Yes or No
Were musculoskeletal topics taught in your medical school's curriculum during clerkship?	Yes or No
What estimated percentage of your medical school curriculum was dedicated to musculoskeletal knowledge?	0-100%
What aspect of musculoskeletal knowledge did your medical school curriculum cover? Check all that apply	MSK Disease Treatment, MSK Imaging, MSK Pathology, MSK Physical Examination, Splinting/Casting, Other (specify)

This project was reviewed and approved by the University of Arizona Institutional Review Board (IRB 1611010724, dated November 4, 2022). All participants provided informed consent, and data confidentiality was maintained throughout the study.

## Results

The demographic data of the cohort are presented in Table 2. All 21 participants completed the assessment. When queried about their UME MSK training, participants provided insightful responses. Of those who participated in related MSK rotations, six residents reported completing rotations in orthopedics, while five had experience in sports medicine (SM), and four in physical medicine and rehabilitation (PMR). Additionally, one resident had undertaken a rotation in rheumatology. Three residents mentioned additional rotations they deemed relevant, including two in osteopathic principles and practices (OPP) and one in wilderness medicine. 

**Table 2:** Demographic Data of the Respondents

Program and Degree Breakdown	Percentage	N (of 21)
Medical School Program Location		
United States	100%	21
West	61.9%	13
Midwest	19.0%	4
Northwest	14.3%	3
South	4.8%	1
Medical Degree		
Allopathic	66.7%	14
Osteopathic	33.3%	7
Residency Program		
Community Campus	28.6%	6
Academic Campus	71.4%	15

Information regarding UME MSK curricula was collated (Table 3), which revealed that 20 out of 21 residents received MSK education during medical school. Among them, 19 reported MSK topics being covered during pre-clerkship, and 15 during clerkship. The estimated proportion of curriculum dedicated to MSK knowledge ranged from 0% to 35%, with a median of 15% and an average of 14.0%. Interestingly, one participant reported not receiving MSK education during medical school but had exposure to SM during their training. Another resident, who claimed no MSK education during pre-clerkship, had a PMR rotation where the MSK curriculum was covered. Among the five residents who reported no MSK education during clerkship, one had rotations in both orthopedics and SM, two in orthopedics only, while the remaining two did not specify any MSK-related rotations.

**Table 3:** Musculoskeletal Medical School Curriculum-Related Responses *The one participant who responded “no” to MSK topics being taught in UME also reported participating in a sports medicine rotation in clerkship MSK: musculoskeletal; UME: undergraduate medical education

UME MSK Curriculum	Percentage	N (of 21)
UME MSK Topics Taught	95.2%	20*
MSK Topics Taught in Pre-clerkship	90.5%	19
MSK Topics Taught in Clerkship	71.5%	15
MSK-Associated Rotations		
Orthopedics	28.6%	6
Sports Medicine	23.8%	5
Physical Medicine and Rehabilitation	19.0%	4
Rheumatology	4.8%	1
Osteopathic Principles and Practices	9.5%	2
Wilderness Medicine	4.8%	1
Total Estimated Percentage of MSK-Dedicated Curriculum		
Average	14.0%	
Median	15%	
MSK Knowledge Domains		
MSK Imaging	76.2%	16
MSK Disease Treatment	66.7%	14
MSK Pathology	85.7%	18
MSK Physical Examination	90.4%	19
Splinting and Casting	38.1%	8
Osteopathic Principles and Practices	14.3%	3

Regarding specific aspects of MSK knowledge covered in their medical school curricula, 16 residents mentioned training in MSK imaging, 14 in MSK disease treatment, 18 in MSK pathology, 19 in MSK physical examination, and eight in splinting and casting. Notably, three of seven osteopathic interns highlighted OPP as additional MSK training in their curriculum. 

The 50-question MEAT was administered to all incoming resident physicians during intern boot camp with 100% compliance in the pretest. The average pretest score for all participants was 29.9, with a median of 30. On average, participants took 32 minutes to complete the assessment.

## Discussion

The feasibility study of MEAT demonstrated a 100% response rate, indicative of successful implementation. Previous online MSK studies have also shown reasonable response rates, though never reaching 100% ​[18]. The high response rate was attributed to the in-person administration during orientation compared to remote administration. The absence of any reported difficulties in accessing, completing, or understanding MEAT indicates that it is feasible that participants will complete subsequent assessments. The mean and median participant scores of 60% indicated minimal variability in responses. While no passing score has yet been established for MEAT, the obtained scores are below conventional passing thresholds, which aligns with the expectation for participants who had not yet undergone any GME activities. Furthermore, the participants took an average of 32 minutes to complete the test, and hence sufficient time should be allocated for its administration. Overall, these findings support the feasibility and potential utility of MEAT as an efficient and accessible tool for assessing MSK knowledge among incoming resident physicians in EM programs.

All participants had documented UME MSK education, with a median of 15% of the total curriculum being dedicated to MSK education. This closely aligns with the estimated 20% prevalence of MSK complaints seen annually in primary care and ED visits. However, the content and clinical relevance of UME MSK education varied significantly, ranging from 0 to 35% of the total curriculum, reflecting variability in MSK knowledge domains and clinical rotations. Although the UME curriculum must fit within the general competencies framework required by accrediting bodies such as the Liaison Committee on Medical Education (LCME) ​[19]​ or the American Osteopathic Association Commission on Osteopathic College Accreditation (COCA) ​[20], there are no specific guidelines on the MSK education content or the phase of UME in which it should be taught. Without such guidance, there is no standardization. Without standardization, tests such as the FB-MSK, MSK30, and MEAT may have to remain specialty-specific rather than generalizable to any medical student.

Following the successful feasibility study, our next step involves leveraging MEAT to establish multiple benchmark data points throughout EM residency training. Through a multi-institutional study involving various EM programs, we aim to generate comprehensive benchmarks for incoming and outgoing EM resident physicians. Educators can use these benchmarks to evaluate the efficacy of their programs on a broader scale. Additionally, they can assess the impact of a specific intervention, such as an MSK rotation or workshop, on resident MSK knowledge. The availability of these data points will enable educators to implement targeted interventions tailored to individual residents or entire programs and drive continuous improvement in MSK education and patient care outcomes within the field of EM.

This feasibility study has several limitations. The small sample size, comprising 21 participants from a single institution, limits the generalizability of the findings. The small sample size also reduces the statistical power to detect significant differences and may not accurately represent the broader population of EM residents across different institutions. Additionally, the study was conducted at a single institution, which may introduce site-specific biases and limit the applicability of the results to other settings with different educational environments and resources. Furthermore, the study did not establish a passing score for the MEAT, meaning that the current scores cannot be definitively interpreted as indicating competency or deficiency in MSK knowledge. We also did not assess the residents' perceived ease of use or satisfaction with the MEAT after completion. Feedback from participants regarding the usability and relevance of the assessment could provide valuable insights for future iterations and broader implementation. As a feasibility study, the primary goal was to assess the practicality and initial response to the MEAT rather than to evaluate its effectiveness comprehensively. Future studies with larger, more diverse cohorts and multi-institutional participation are necessary to validate the MEAT and establish its efficacy in assessing and improving MSK knowledge among EM residents.

## Conclusions

This study underscores the documented shortcomings in MSK knowledge within both undergraduate and graduate medical education, including EM. EM residency programs have attempted to address this gap through various educational methods; however, existing validated MSK assessments are neither specific to EM nor user-friendly in terms of administration and grading. The novel MEAT has proven feasible for administration, offering a standardized method for assessing EM-specific MSK knowledge. The successful implementation and high response rate in this study highlight MEAT's potential to identify baseline MSK knowledge and areas needing improvement. While broader validation across multiple institutions is necessary to enhance the generalizability of MEAT, it already fills a significant void in current EM MSK assessments. By identifying specific deficiencies, MEAT enables educators to implement targeted, trainee-specific, or program-wide curricular interventions. This tool is poised to become an integral part of EM residency programs, ultimately improving MSK competency and patient care outcomes in this crucial area of medicine.

## Figures and Tables

**Figure 1:**
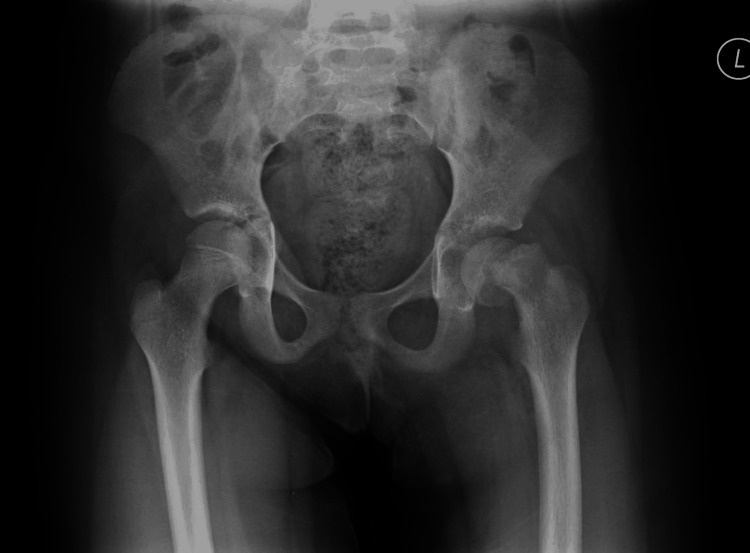
Anteroposterior Radiograph of the Pelvis Case courtesy of Hani Makky Al Salam, Radiopaedia.org

**Figure 2:**
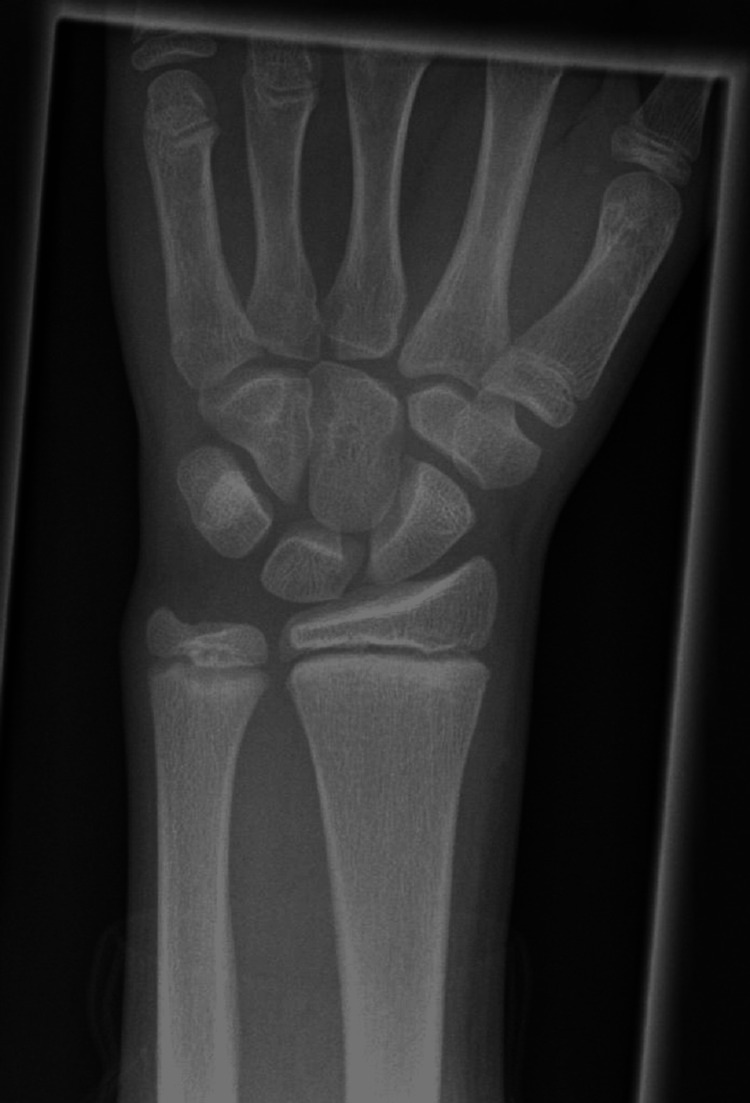
Anteroposterior Radiograph of the Wrist Image courtesy of Ian Bickle, Radiopaedia.org

**Figure 3:**
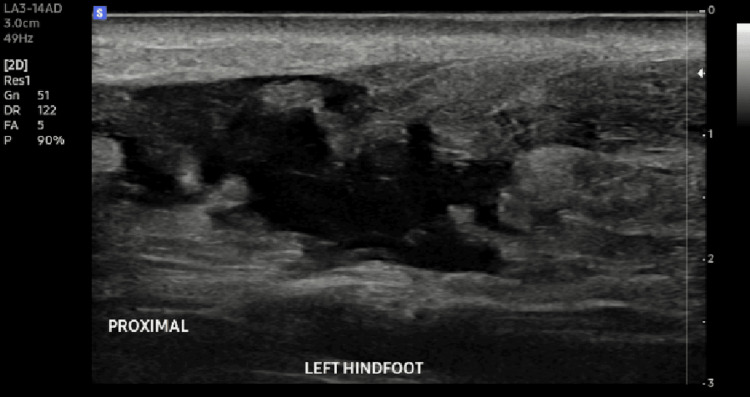
Longitudinal Ultrasound View of the Left Hindfoot Image courtesy of Matthew Negaard

**Figure 4:**
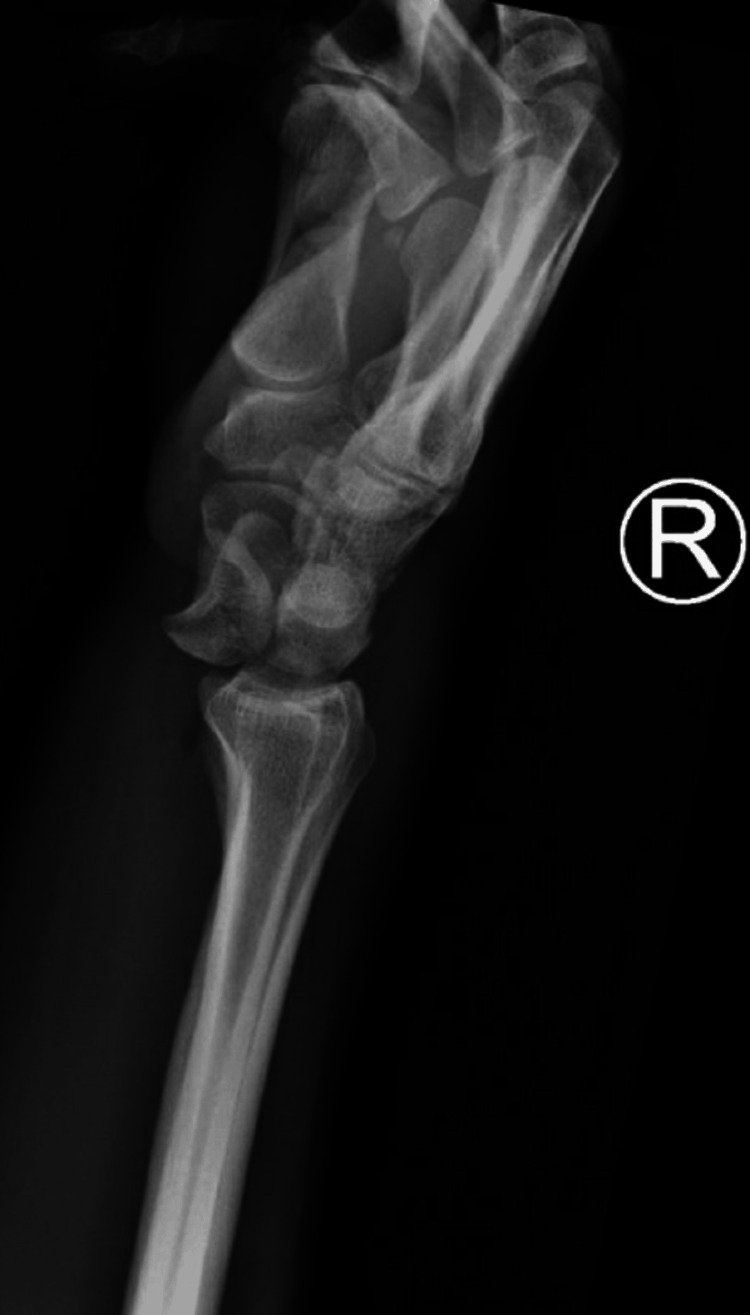
Lateral Radiograph of the Wrist Image courtesy of Will Denq

**Figure 5:**
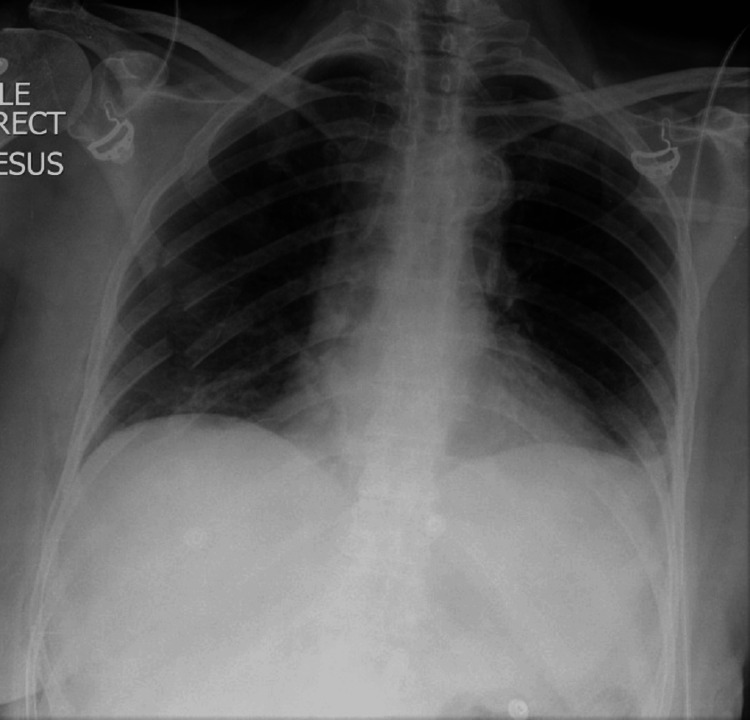
Anteroposterior Radiograph of the Chest Case courtesy of Ian Bickle, Radiopaedia.org

**Figure 6:**
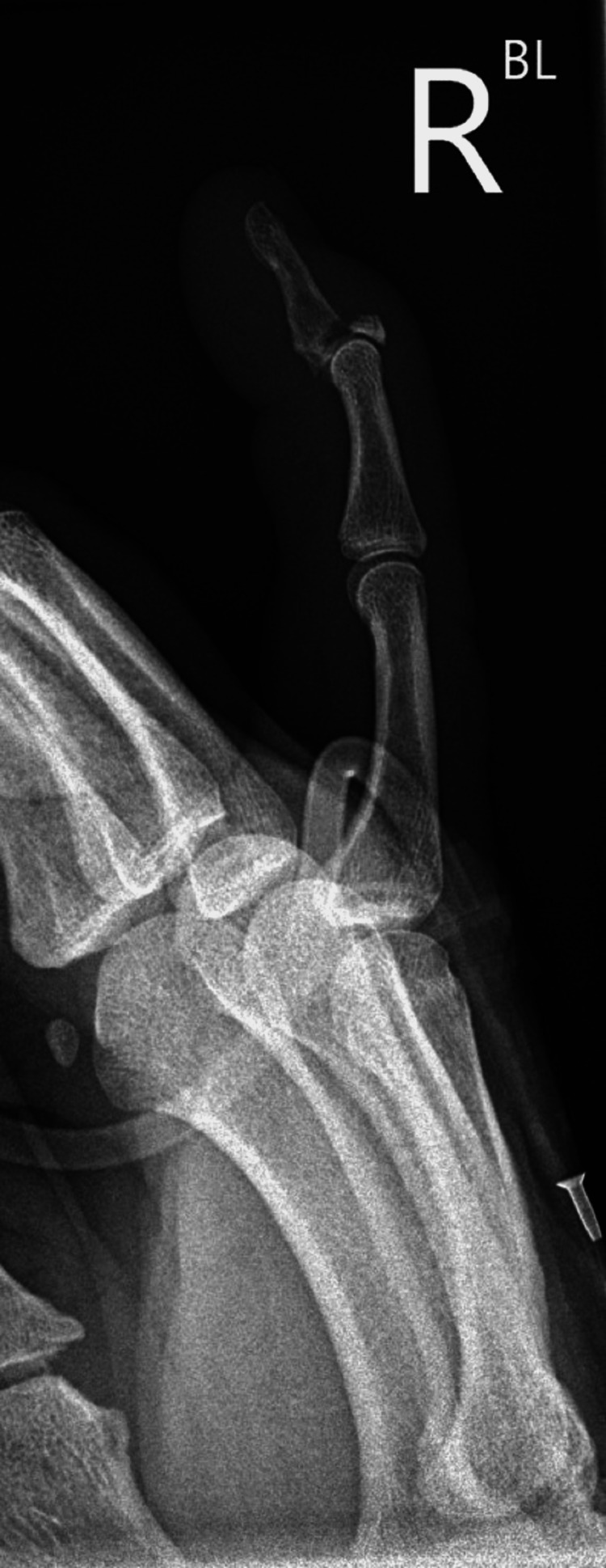
Lateral Radiograph of the Digit Case courtesy of Andrew Taylor, Radiopaedia.org

**Figure 7:**
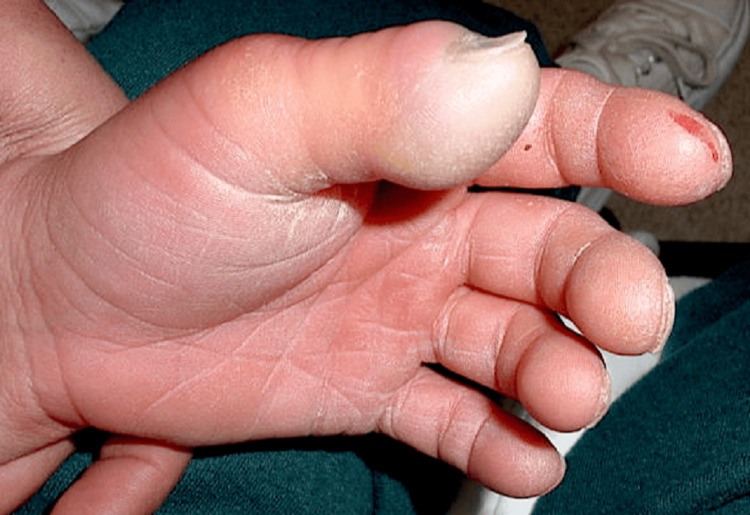
Photo of the Affected Digit Image courtesy of Adam Rosh, Rosh Review

**Figure 8:**
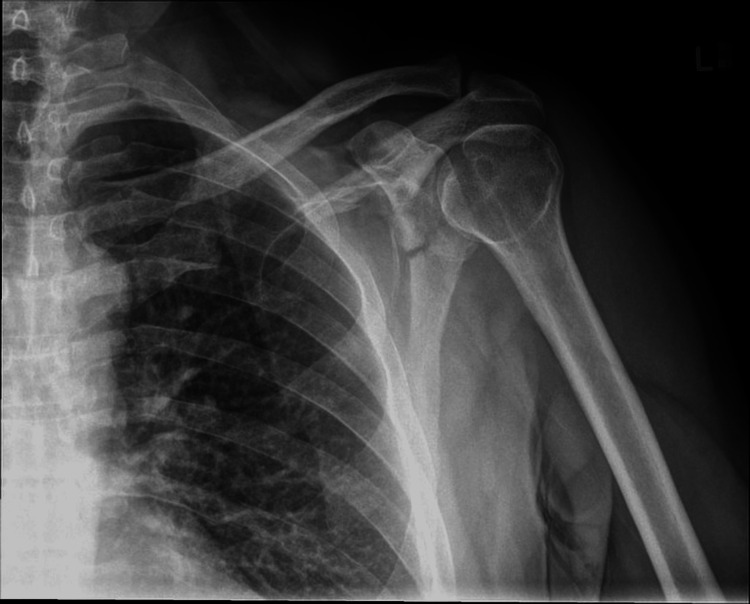
Anteroposterior Radiograph of the Shoulder Case courtesy of Henry Knipe, Radiopaedia.org

## Appendices

**Table 4:** Musculoskeletal Emergency Medicine Assessment Tool* *[14]

Question	Answer A	Answer B	Answer C	Answer D	Figure
1. A 74-year-old male presents with a distal radius fracture. You attempt to reduce it in the emergency department. Which of the following best describes your reduction technique?	Apply an axial load, restore alignment, and then recreate the mechanism of injury	Apply an axial load, recreate the mechanism of injury, and then restore alignment	Apply traction, recreate the mechanism of injury, and then restore alignment	Apply traction, restore alignment, and then recreate the mechanism of injury	N/A
2. You obtain an elbow X-ray on a pediatric patient and are concerned about a supracondylar fracture. There is no posterior fat pad sign on the lateral view. What additional radiographic feature helps reduce the likelihood of a supracondylar fracture?	The anterior humeral line should intersect the middle third of the capitellum	The anterior humeral line should intersect the posterior third of the capitellum	The posterior humeral line should intersect the middle third of the capitellum	The posterior humeral line should intersect the posterior third of the capitellum	N/A
3. A 22-year-old college student presents to your emergency department for evaluation. He has a fracture of the 5th metacarpal neck on an x-ray. What is the correct name and splint for this injury?	Radial gutter splint	Sugar tong splint	Thumb spica splint	Ulnar gutter splint	N/A
4. You are evaluating a 22-year-old patient with a fall on an outstretched hand. The patient has anatomical snuff box tenderness. Radiographs are negative. Which management step is most appropriate?	Compression dressing	Sugar tong splint	Thumb spica splint	Ulnar gutter splint	N/A
5. You are evaluating a middle-aged male with right shoulder pain. He states he fell backward on a ladder and felt a pop. You are suspicious of glenohumeral dislocation. Which plain film view would be most appropriate to accurately diagnose an anterior dislocation?	Anterior-posterior view	Axillary view	Internal rotation view	Zanca view	N/A
6. You are evaluating a 54-year-old male with a significant knee injury after a motorcycle crash. He reports he had an anterior translation of the tibia prior to it “popping back in”. On exam, he has a 1+ DP and PT pulse, decreased compared to the contralateral side, what is the most appropriate next step in management?	If the ankle-brachial index is less than 0.9, discharge home with urgent outpatient orthopedics follow-up	Perform computed tomography angiography, consult Orthopedics, and consult Vascular surgery if vascular injury is found	Place in knee immobilizer, discharge home with urgent outpatient orthopedics follow-up	Serial exams for 2 hours, if neurovascular exam remains the same, discharge with urgent outpatient orthopedics follow-up	N/A
7. A patient presents with right ankle pain after a significant fall. He is found to have a posterior ankle dislocation. What is the most appropriate technique used to reduce these injuries?	Extend the knee, dorsiflex the ankle, apply axial traction on the foot, and then simultaneously posterior pressure on the calcaneus	Extend the knee, plantar-flex the ankle, apply axial traction on the foot, and then simultaneously apply lateral pressure on the calcaneus	Flex the knee, plantar-flex the ankle, apply axial traction on the foot, and then simultaneously apply anterior pressure on the calcaneus	Flex the knee, plantar-flex the ankle, apply axial traction on the foot, and simultaneously apply medial pressure on the calcaneus	N/A
8. A 22-year-old male presents to the ED after falling and striking his head while intoxicated. There is concern for cervical spine injury. Which of the following is considered a stable cervical fracture?	Bilateral facet dislocation	Flexion teardrop fracture	Jefferson fracture	Wedge fracture	N/A
9. In addition to your usual Anterior-Posterior and Lateral views, what other x-ray view can be most useful when evaluating for C1 burst fracture?	Extension view	Flexion view	Odontoid view	PA oblique view	N/A
10. You are evaluating a 23-month-old female who presents with a limp on weight-bearing. Her mom states she has been limping for a few days. She denies any trauma but endorses recent upper respiratory infection symptoms. She is currently afebrile. Which of the following diagnoses is most likely?	Septic arthritis	Slipped capital femoral epiphysis	Toddler’s fracture (tibial shaft fracture)	Transient synovitis of the hip	N/A
11. A 13-year-old obese male presents to the ED with left hip and knee pain progressing over the last month. The radiographs obtained are shown below (Figure 1). What is the most likely diagnosis? Figure 1: Case courtesy of Hani Makky Al Salam, Radiopaedia.org	Legg-Calve-Perthes disease (LCPD)	Lesser trochanter avulsion fracture	Normal variant	Slipped Capital Femoral Epiphyses (SCFE)	Figure 1
12. A 60-year-old female presents with acute on chronic low back pain. She has a history of IV heroin use. Lately, she has been having fevers, fatigue, and difficulty walking. Vital signs are T: 39, BP: 90/55, P: 122, R: 26. What is the best initial step?	Intravenous antibiotics	Lumbar puncture	Magnetic resonance imaging	Neurosurgical consultation	N/A
13. A 50-year-old male presents with acute on chronic low back pain. He now feels pain down his both legs and they feel weak. Bedside ultrasound shows a post-void residual of 300 mL. What is the most appropriate disposition?	Emergent neurosurgery consult for surgical decompression	Follow up with neurosurgery for outpatient surgical intervention	Intravenous antibiotics and admission	Outpatient physical therapy	N/A
14. A patient presents to the ED for a hot and swollen ankle. On examination, they have pain with a passive range of motion. You perform an arthrocentesis. The results of the fluid analysis show 120,000 WBC, 96% neutrophils, and negatively birefringent crystals in needle shapes. What is the diagnosis and the next step in management?	Gout, discharge	Osteoarthritis, ibuprofen	Pseudogout, prednisone	Septic arthritis, consult orthopedics	N/A
15. Patient presents to the emergency department with swelling over the left olecranon. They have no pain with pronation/supination of the elbow. The overlying skin is unremarkable with no erythema or increased warmth. What is the next best step?	I&D for concern of abscess	Perform a diagnostic tap to rule out septic arthritis	Perform a therapeutic tap to drain all fluid from this collection	Recommend compression and anti-inflammatory treatment	N/A
16. You are evaluating an 8-year-old athlete who rolled his ankle playing basketball. On exam, there is exquisite tenderness to the lateral malleolus. Radiographs show a fracture line only through the epiphysis and does not cross the physis. Which Salter-Harris classification would this child have?	Salter-Harris I	Salter-Harris II	Salter-Harris III	Salter-Harris IV	N/A
17. A 10-year-old female presents with wrist pain following a FOOSH off her bicycle. On exam, there is swelling and tenderness over the physis of the distal radius. There is no tenderness to the anatomic snuff box. The radiograph is shown below (Figure 2). What would be the suspected diagnosis and treatment? Figure 2: Image courtesy of Ian Bickle, Radiopaedia.org	Salter-Harris I fracture; splint	Salter-Harris V fracture; splint	Scaphoid fracture; splint	Sprain; ACE wrap	Figure 2
18. A 33-year-old male presents to your ED with leg pain. He was seen yesterday and diagnosed with a tibia fracture, placed in a splint, and discharged. He returns due to severe pain. Even after removing the splint, he is in severe pain at rest, pain with passive stretch, decreased sensation, and his anterior compartment is tense. Your nearest hospital with orthopedics is 6 hours away. Which would be the most appropriate treatment plan?	Elevate the leg to decrease swelling, pain control with regional block, obtain compartment pressures	Intravenous pain medication, consider fasciotomy, transfer to a hospital with orthopedics	Keep the leg in a dependent position to increase perfusion, pain control, obtain compartment pressures	Pain control with regional block, transfer to hospital with orthopedics	N/A
19. A patient presents to the ED after a long bone fracture. In which of the following presentations would you be most concerned for acute compartment syndrome?	A patient with a crush injury to the forearm with a delta pressure of 35 mmHg	A patient with a femur fracture and diastolic blood pressure of 50 and intra-compartment pressure of 25 mmHg	A patient with fibula fracture and delta pressure of 50 mmHg	A patient with a tibia fracture with anterior compartment pressure of 20 mmHg	N/A
20. A patient presents after being struck by a vehicle while crossing the street. He is reporting severe back pain. Which of the following thoracic spine fractures would most likely be unstable?	Burst fracture	Clay-Shoveler fracture	Compression fracture	Transverse process fracture	N/A
21. You are evaluating a 33-year-old female in the trauma bay status post-high-speed MVC. She has multiple spinal fractures. Which of the following fractures should increase your index of concern for concomitant intra-abdominal injury?	Burst fracture	Chance fracture	Clay-Shoveler fracture	Compression fracture	N/A
22. A patient presents to the Emergency Department after a motor vehicle accident. She reports abdominal and pelvic pain. What pelvic fracture is considered unstable?	Avulsion fracture of the anterior iliac spine	Iliac wing fracture	Superior and inferior pubic ramus fracture	Vertical shear fracture	N/A
23. A patient presents to the Emergency Department after a motor vehicle accident. He reports abdominal and pelvic pain. His blood pressure is 86/55. Examination reveals gross pelvic instability. Where should a pelvic binder be centered over?	Greater trochanters	Inferior iliac spine	Iliac crest	Superior iliac spine	N/A
24. A patient presents to the Emergency Department after a tractor rollover. He has a tibia fracture with a 15 cm wound with significant tissue loss, that will require tissue flap to cover. The wound is grossly contaminated with dirt. What is the most appropriate antibiotic regimen?	Cefazolin and ciprofloxacin	Cefazolin, penicillin G, and gentamicin	Ceftriaxone and clindamycin	Ceftriaxone and Gentamicin	N/A
25. A 52-year-old male with an open tibia fracture presents to the ED. The tibial fracture is protruding through the skin. How should the open fracture be managed?	Consult orthopedics for surgical intervention	Place a wet dressing, consult orthopedics for surgical intervention	Start IV antibiotics, reduce fracture, place a splint, consult orthopedics for surgical intervention	Wash out the wound in the ED, reduce the fracture, place a splint, close the wound, and discharge	N/A
26. A patient presents to the ED after a fall off their bicycle. The workup shows a midshaft humeral fracture. The patient has an associated neurological deficit, what examination technique would show the expected deficit?	Finger abduction	Finger flexion	Thumb adduction	Wrist extension	N/A
27. You are evaluating a patient with atraumatic knee pain and no systemic symptoms. He has normal vital signs. He has an effusion and a history of significant alcohol use. Which of the following fluid analysis is expected?	5,000 WBC, neutrophils 60%, positively birefringent rhomboid-shaped crystals	5,000 WBC, neutrophils 75%, no crystals	15,000 WBC, neutrophils 50%, negatively birefringent needle-shaped crystals	100,000 WBC, neutrophils 97%, no crystals	N/A
28. A 40-year-old male with a history of IV substance abuse presents with left calf tenderness and swelling. He reports fatigue and subjective fevers. Temperature 101.4 F. Heart rate is 98. WBC 12.5, ESR 65, CRP 40. CK is within normal limits. A radiograph of the tibia/fibula demonstrates soft tissue swelling and gas formation. What is your next step?	Emergently consult for surgical evaluation	Obtain an MRI of the lower extremity	Obtain a CT of the lower extremity	Perform a finger test	N/A
29. A patient presents to the ED 3 weeks after total hip arthroplasty, unable to bear weight on the leg. X-ray shows anterior hip dislocation. What additional diagnostic testing should be performed in addition to reducing the hip and consulting orthopedic surgery?	Computed tomography of the hip	Laboratory workup to rule out infection	Magnetic resonance imaging of the hip	Ultrasound of the hip	N/A
30. A patient presents with a chronic foot ulcer. Which imaging modality is most sensitive and specific for identifying acute osteomyelitis?	Computed tomography	Magnetic resonance imaging	Plain film radiographs	Ultrasound	N/A
31. You are evaluating a 44-year-old male who had sudden ankle pain and felt a pop while playing basketball. You perform an ultrasound of the posterior ankle that looks like the below (Figure 3), what is the diagnosis? Figure 3: Image courtesy of Matthew Negaard	Achilles tendon rupture	Calcaneal spur	Ganglion cyst	Gastrocnemius strain	Figure 3
32. A 54-year-old male presents with knee pain. He states that he was playing basketball when he felt a sudden knee pain after jumping and landing. He is unable to extend his knee against gravity. Given the most likely diagnosis, what is the most appropriate form of immobilization for this patient?	Hinged knee brace	Knee immobilizer	Stirrup splint	Tall walking boot	N/A
33. You evaluate a 42-year-old male roofer with elbow pain status post fall off the roof. After confirming he is neurovascularly intact, you obtain X-rays that reveal a posterior elbow dislocation. You successfully reduce it. What immobilization method is most appropriate for a reduced, posterior elbow dislocation?	Posterior long arm splint	Shoulder immobilizer	Shoulder sling	Sugar tong	N/A
34. A patient presents to the ED after a FOOSH injury. X-ray is obtained and shown below (Figure 4). What is the most appropriate next step in management? Figure 4: Image courtesy of Will Denq	Closed reduction and splinting with close hand surgery follow-up	Magnetic resonance imaging of the wrist	Short arm cast	Thumb spica splint with repeat x-rays in 1 week	Figure 4
35. You have a 3-year-old in your pediatric emergency department who is not using their arm. You suspect a nursemaid's elbow. What is the specific injury associated with this diagnosis?	Apophysitis of the olecranon	Dislocation of the radial head	Salter-Harris I fracture	Subluxation of the radial head	N/A
36. A 21-year-old male presents with chest pain after he was struck by a speeding sedan. On examination, you notice paradoxical movement of the right chest wall. Vital signs are significant for SpO2: 89%. Point of care ultrasound shows a positive slide sign in all fields. There are no open wounds on the chest wall noted. Chest radiograph below (Figure 5). What is your immediate next step? Figure 5: Case courtesy of Ian Bickle, Radiopaedia.org	Perform a CT of the chest	Place a chest tube	Place the patient on supplemental oxygen	Place a temporary splint over the injured area to assist with breathing	Figure 5
37. A 33-year-old prison inmate presents with right shoulder pain. He is diagnosed with a shoulder dislocation, which is subsequently reduced. What nerve is most commonly injured in an anterior shoulder dislocation?	Axillary	Median	Radial	Ulnar	N/A
38. A 25-year-old industrial painter presents to the ED at his boss’s behest after accidentally running the tip of his index finger under a paint sprayer. He denies any pain or discomfort and has a full range of motion of his finger. What is the most appropriate next step in management?	Bedside I&D with a digital block	Discharge with oral antibiotics and outpatient follow-up with PCP	Intravenous antibiotics and observation x 24 hours	Urgent hand surgery consultation	N/A
39. You are evaluating a 17-year-old football player with right knee pain and an obvious deformity following a collision. You suspect a lateral patellar dislocation. Which of the following best describes the reduction technique?	Gently extend the knee while applying laterally directed pressure to the patella	Gently extend the knee while applying medially directed pressure to the patella	Gently flex the knee while applying laterally directed pressure to the patella	Gently flex the knee while applying medially directed pressure to the patella	N/A
40. You are evaluating a 34-year-old patient with hand pain. They state that they had a puncture wound while swimming in the ocean. They present to the ED with worsening pain and you are suspicious of flexor tenosynovitis. Which of the following correctly identifies one of the Kanavel signs?	A finger is held in a slight extension	Localized swelling of the pulp of the fingertip	Pain with passive flexion	Tenderness over the flexor tendon	N/A
41. A patient presents to the ED after a bicycle accident where he landed on the left shoulder. His x-ray shows a mid-clavicle fracture with 1 cm of displacement and no skin tenting. What is the appropriate disposition?	Attempt closed reduction, place in arm immobilizer, non-weight bearing to left upper extremity, and follow up with orthopedic surgery in 4 weeks	Place in a sling, non-weight bearing to the left upper extremity, and follow up with PCP/sports medicine in 1 week	Place in coaptation splint, non-weight bearing to left upper extremity, and follow up with PCP/sports medicine in 1 week	Urgent orthopedics consult for surgical intervention	N/A
42. A football player presents to the ED with diffuse muscle pain after starting summer training camp 3 days ago. He notes tea-colored urine. What is the most likely finding on urinalysis?	(-)blood, full field RBCs, (+)myoglobin	(-)blood, 0 RBCs, (-)myoglobin	(+)blood, full-field RBCs, (+)myoglobin	(+)blood, 0 RBCs, (+)myoglobin	N/A
43. A baseball player presents to the ED after sliding into a base and is unable to fully straighten his finger at the DIP joint. His x-ray is as shown (Figure 6). What is the most appropriate next step in management? Figure 6: Case courtesy of Andrew Taylor, Radiopaedia.org	Buddy tape and follow-up with ortho as an outpatient	Closed reduction and splinting	Splint DIP joint in full extension	Urgent hand surgery consultation	Figure 6
44. You are evaluating a patient with shoulder pain after a motor vehicle crash. You suspect a clavicular injury and imaging is pending. What clavicular injury is most likely to have secondary life-threatening injuries?	Grade 2 Acromioclavicular joint dislocation	Midshaft clavicular fracture	Posterior sternoclavicular joint dislocation	Unstable distal clavicle fracture	N/A
45. A patient presents to the ED with the following hand findings (Figure 7). What is the best treatment for this condition? Figure 7: Image courtesy of Adam Rosh, Rosh Review	Longitudinal incision along the radial aspect	Longitudinal incision over the pulp of the thumb	Oral antibiotics	Warm soaks and elevation	Figure 7
46. A 19-year-old college gymnast presents with left foot pain and swelling. He states he was tumbling and came down awkwardly on his foot. You are concerned for a Lisfranc injury. Which immobilization is most appropriate?	Compression bandage, non-weight bearing	Posterior long leg, weight-bearing	Pneumatic boot, weight-bearing	Post-op shoe, non-weight bearing	N/A
47. A 17-year-old patient dove into the shallow end of a pool, striking his head on the cement floor. He noted immediate midline neck pain but was neurovascularly intact. A CT scan of the cervical spine reveals a vertebral body fracture of C3. Which of the following suggests that this fracture is stable?	55% loss of vertebral body height	Minimal displacement of the posterior column	55% spinal canal narrowing	Posterior vertebral retropulsion	N/A
48. An intoxicated patient is brought in by police to your ED after a fight with another bar patron. On exam, you note a deep laceration over the dorsal aspect of his 4th MCP joint, with concern for communication with the joint. He has normal ROM of his fingers. What is the most appropriate next step in management?	Bedside irrigation and closure	Hand surgery consultation	Leave open and place in an ulnar gutter splint	Leave open and discharge with oral antibiotics	N/A
49. A 60-year-old male presents with left shoulder pain following a fall from a standing height. He is neurovascularly intact and his trauma evaluation only demonstrates this isolated injury on radiograph (Figure 8). What is the optimal treatment for the represented injury? Figure 8: Case courtesy of Henry Knipe, Radiopaedia.org	Coaptation splint and urgent outpatient orthopedic follow-up	Shoulder immobilizer and emergent orthopedic consultation	Shoulder immobilizer and standard outpatient orthopedic follow-up	Shoulder sling and urgent outpatient orthopedic follow-up	Figure 8
50. A patient presents to the ED complaining of back pain. Which of the following is the most frequent cause of discitis?	Hematogenous spread	Localized spread	Recent surgery/procedure	Trauma	N/A

The figures mentioned in the table are presented below (Figures 1-8). 
